# Field Programmable Gate Array-Based Smart Switch to Avoid Inrush Current in PV Installations

**DOI:** 10.3390/s24041121

**Published:** 2024-02-08

**Authors:** Gerardo de J. Martínez-Figueroa, Felipe Córcoles, Santiago Bogarra

**Affiliations:** Department of Electrical Engineering, Escola Superior d’Enginyeries Industrial, Aeroespacial i Audiovisual de Terrassa, Universitat Politècnica de Catalunya, 08222 Terrassa, Spain; gerardo.de.jesus.martinez@upc.edu (G.d.J.M.-F.); felipe.corcoles@upc.edu (F.C.)

**Keywords:** grid-connected PV systems, transformer, saturation, smart switch, FPGA, inrush current

## Abstract

This paper introduces an FPGA-based implementation of a smart switch designed to avoid inrush currents occurring during the connection of single-phase transformers utilized in grid-connected photovoltaic (PV) systems. The magnitude of inrush currents is notably impacted by the residual flux within the transformer core and the precise moment of energization relative to the wave cycle. Alternative methods frequently hinge on intricate procedures to estimate residual flux. This challenge is adeptly circumvented by the innovative smart control system proposed herein, rendering it a cost-effective solution for grid-connected PV systems. The proposed solution for mitigating inrush current remains effective, even in the face of challenges with current and voltage sensors. This resilience arises from the system’s ability to learn and adapt by leveraging information acquired from the network.

## 1. Introduction

Field Programmable Gate Arrays (FPGAs) are a top pick for industrial, power, and Internet of Things (IoT) applications, thanks to their robust processors, evolving capacity, and cost-effective reconfigurability. Their unique blend of reconfigurability and parallel processing makes them more valuable than microcontrollers, enabling the development of highly efficient hardware architectures with exceptional energy efficiency [1,2,3,4].

Various references discuss FPGA applications in power systems, with highlighted examples [5,6,7,8,9]. In [5], an FPGA-based smart energy meter enables remote monitoring of power systems, adapting to different conditions, including bidirectional power flow in distributed generation with photovoltaic systems. The implementation in an FPGA of advanced signal processing techniques, such as orthogonal empirical mode decomposition, is proposed in [6] to identify power quality disturbances (PQDs) in real time. In [7], PQD detection relies on the Hilbert–Huang transform on an FPGA. For the same purpose (detect and classify PQD), Ref. [8] presents an FPGA-based smart sensor that integrates higher-order statistic processing cores, while Ref. [9] proposes a portable FPGA-based system that achieves PQD recognition and classification through discrete wavelet transform, mathematical morphology, decomposition of singular values, and statistical analysis. On the other hand, Ref. [10] details an FPGA implementation of a power quality analyzer.

The FPGA implementation of a methodology based on statistical time features and support vector machines (SVMs) for the diagnosis of short-circuited turn faults is presented in [11]. For the diagnosis methodology, 19 indicators from the transformer vibration signals are computed; then, the most discriminant features are selected. Finally, a support vector machine classifier is employed to achieve the diagnosis automatically.

Other papers focus on electrical controls [12,13,14]. Ref. [12] uses an FPGA in the loop for sliding mode control in wind energy’s Doubly Fed Induction Generator (DFIG) modeling. In [13], FPGA enables finite state-predictive direct current control (FS-PDCC) for power converters. Ref. [14] introduces an FPGA-based Grid Friendly Appliance (GFA) controller for grid frequency monitoring and load disconnection.

In [15,16], novel approaches for the real-time simulation of power converters in FPGAs are proposed, while Ref. [17] introduces a sub-microsecond-level real-time simulation method for microgrids. The case studies demonstrate that the proposed simulation methods consume significantly fewer FPGA resources than traditional approaches.

Researchers have optimized overcurrent relays using FPGA technology for high-speed protective relays in smart grids [18,19,20,21]. In [18], an ANN-based flexible over-current relay on an FPGA achieves an ultra-low processing time and adaptive remote settings. Ref. [19] details an FPGA-based overcurrent relay with concurrent sense-process-communicate cycles and an FFT filter for precise current component isolation. Ref. [20] introduces a real-time, low-latency hardware digital distance protective relay on FPGA for efficient data throughput in high-frequency sampled data. Ref. [21] implements an adaptive Mho characteristic on an FPGA board, using phasor estimation errors for swift computational speeds and integrating Ethernet-based protocols to validate relay performance in digital substation environments.

DC grids are gaining popularity for their efficiency, especially with DC-based renewables like solar and wind power. This is useful for applications like electric vehicles and DC electric railways as it eliminates the need for an AC conversion, improving the overall efficiency [22,23]. In [22], a DC circuit breaker for high-voltage DC transmission systems reduces surge voltage during current clearing using semiconductor devices and a freewheeling diode. FPGA-based controllers are used for converters and DC breakers. In low-voltage DC microgrids during short-circuit faults, a solid-state circuit breaker’s safety depends on effective snubber overvoltage suppression. Reference [23] introduces a specific snubber design method using FPGA-based controllers for three tested snubbers.

FPGA controllers play a crucial role in PV systems due to their adaptability, robust processing power, parallel processing capabilities, and flexibility to accommodate changing standards [24,25,26,27]. In [24], a DSTATCOM model with an ANN controller addresses power quality issues related to current by implementing an online learning-based algorithm suitable for balanced non-linear loads. Validation is conducted through experiments using an FPGA controller. In [25], power-sharing control (PSC) for a solar PV system integrated into a low-voltage DC nano-grid is implemented on an FPGA. Ref. [26] introduces a reconfigurable FPGA implementation for Maximum Power Point Tracking (MPPT) in PV systems, utilizing a fuzzy logic-based controller effective under variable irradiance and temperature conditions. Ref. [27] presents an FPGA-based MPPT implementation using a dual Kalman filter for real-time estimation of the settling time of the entire system.

Energizing transformers can induce a significant non-sinusoidal inrush current, especially with residual flux, leading to mechanical stress, relay tripping, and voltage sags affecting power quality [28,29,30,31]. Resonance risks, causing temporary overvoltages (TOVs) and potential transformer winding failures, are explored in [32,33,34]. Ref. [28] introduces methods for evaluating and mitigating voltage sags during energization. Refs. [29,30] investigate the impact of inrush currents on transformer coil mechanical forces using FEM models. In [31], probabilistic distance measure (PDM) is explored to prevent damages from differential protection failures in distinguishing inrush from internal faults. Ref. [32] examines uncertainties affecting resonant overvoltages during transformer energization. Ref. [33] studies switching transient impacts on arresters, proposing preventive measures. Ref. [34] offers analysis tools, using traveling window DFT, to assess the likelihood of overvoltages from inrush currents during offshore transformer startups connected by sub-sea cables.

In [35,36,37], several methodologies are proposed to identify inrush currents in protection schemes and distinguish them from fault currents.

Addressing challenges during energization is crucial for system reliability and equipment protection [38,39,40]. Ref. [38] introduces a simplified phase-controlled switching strategy to reduce high inrush currents in Y-ungrounded transformers. Ref. [39] analyzes inrush current suppression in nuclear plant transformers during no-load closing using a pre-magnetization technique with a small transformer (Pre-T) in series, without the need for closing angle control. Ref. [40] limits inrush current during black starts of medium voltage distribution networks (MV-DNs) through battery energy storage system (BESS) control.

Renewable energy integration, particularly PV transformer energization, can impact power system stability and quality. In [41], the influence of PV transformer energization is analyzed, exploring issues like harmonic resonance and voltage distortion.

In [42,43,44,45], the challenges of inrush currents during PV transformer energization are discussed. Ref. [42] suggests peak instant switching and high-frequency operation to stabilize transformer-integrated PV systems. Ref. [43] proposes a method to minimize inrush current by optimally selecting the wave-energizing instant. Ref. [44] introduces an approach to eliminating magnetization inrush currents and voltage sags in step-up transformers for renewables. Ref. [45] focuses on power converter control, using state feedback and adjusting magnetic flux with an FPGA-generated gate signal to prevent issues during grid fault clearance.

This research introduces a smart switch implemented with FPGA technology to mitigate inrush currents when connecting single-phase transformers in grid-connected PV systems. Unlike methods requiring complex procedures for residual flux estimation, the proposed smart system effectively overcomes this challenge. It provides a robust and adaptive solution, addressing potential problems with current and voltage sensors by taking advantage of their learning and adaptive capabilities based on the acquired information.

Unlike other existing methodologies, which need to acquire voltage and current signals from the transformer continuously, our system requires these measurements only once. Building upon these initial measurements, it calculates the de-energization point-on-wave and the energization point-on-wave, which remain constant through subsequent transformer connections and disconnections. Subsequently, only monitoring the grid voltage is necessary. The inclusion of additional sensors is justified because, while the initial measurements are needed only once, the system becomes more robust by adapting to changes in conditions, such as transformer aging or switch deterioration. In such situations, the de-energization point-on-wave and the energization point-on-wave may experience variations, demonstrating the proposed system’s flexibility and adaptability.

## 2. Theoretical Background

**Figure 1:**
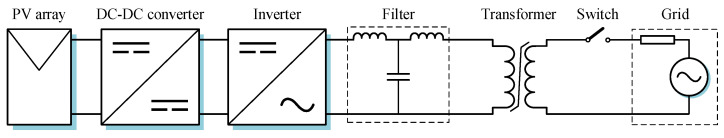
Basic setup of a one-phase grid-tied PV system.

The basic configuration of a single-phase grid-tied PV system incorporating a transformer is shown in Figure 1. The system comprises a solar panel array, a DC–DC converter, a single-phase inverter, an LCL filter, a saturable transformer, and a switch.

The proposal involves disconnecting the PV system first, then disconnecting the transformer from the grid. Doing it the other way around (first disconnecting the grid and then the PV) implies having a lower residual flux (since the transformer is being fed from the low voltage side). In other words, the residual flux also depends on the supply voltage, and the converter topology will affect the voltage drop across the converter. As voltages are measured, if the reverse disconnection process is performed, the residual flux will be determined by the measured voltage, not by the converter topology.

Notably, connecting the transformer to the distribution grid or recovering from faults may result in elevated inrush currents, particularly when the ferromagnetic core is driven into saturation.

### Inrush Current and Residual Flux

**Figure 2:**
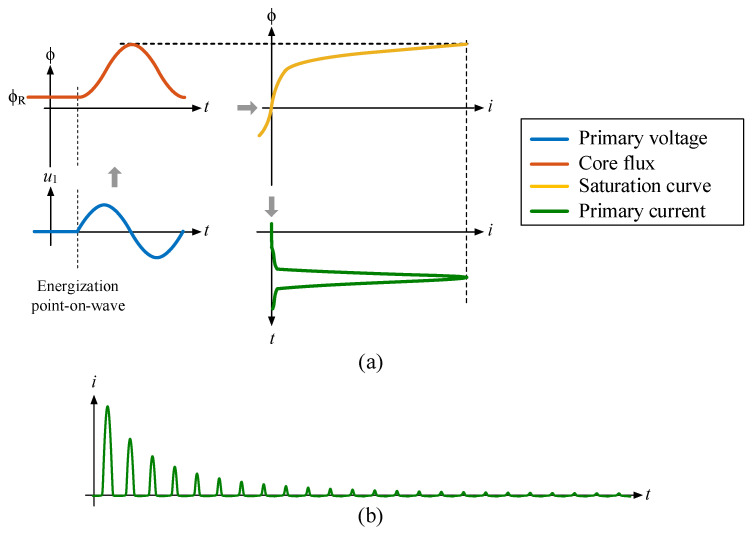
(**a**) Inrush current generation and (**b**) typical inrush current waveform.

Power transformers are designed to operate slightly above the knee point of the saturation (or anhysteretic) curve during steady-state conditions. A slight increase in flux beyond this point results in a noticeable rise in current (known as inrush current), as illustrated in Figure 2a. When a transformer is switched off (de-energized), the iron core of the transformer may retain residual flux due to hysteresis. After being switched on (energized), the maximum theoretical flux peak can reach twice the rated flux peak plus the residual flux (ϕ_R_). The identification of the optimal point-on-wave for energization, a critical factor in mitigating inrush current, depends on the magnitude of residual flux. These two factors, energization point-on-wave and residual flux, are the only controllable variables influencing inrush current.

**Figure 3:**
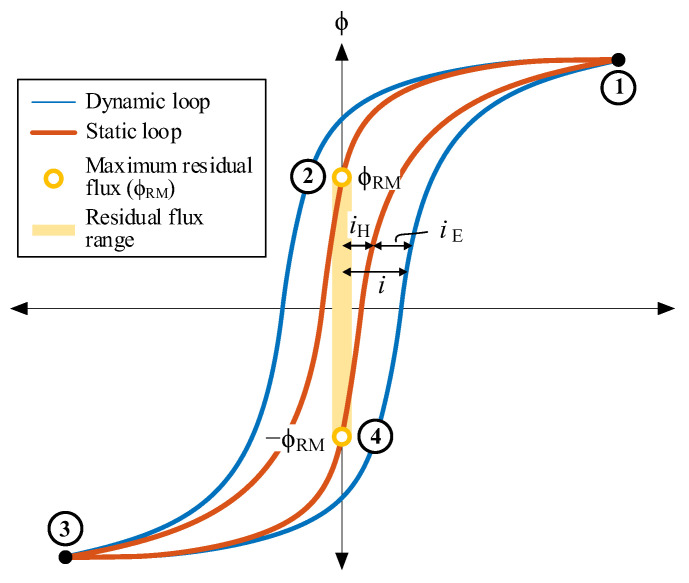
Static and dynamic hysteresis loops and residual flux range.

The iron losses in a transformer core can be classified into three categories: hysteresis losses, classical eddy-current losses, and excess or anomalous losses [46,47]. The hysteresis losses are considered static as their value per cycle is independent of the operating frequency. These losses are proportional to the enclosed area within the static hysteresis loop (depicted by the red line in Figure 3), with evolution contingent on past or historical values. In contrast, eddy losses are considered dynamic as their value per cycle exhibits frequency dependence [48,49].

Static hysteresis is responsible for the residual flux. Since classical eddy losses and excess losses have no impact on residual flux, both elements are collectively categorized as eddy losses in this study. Consequently, the no-load current, denoted as *i*, is the sum of the current attributed to hysteresis losses (*i*_H_) and the current arising from eddy losses (*i*_E_), as illustrated in Figure 3. This yields two distinct loops:

- A static hysteresis loop (ϕ–*i*_H_, red line in Figure 3). This loop is a result of core magnetization and hysteresis losses and cannot be directly measured through the classical no-load test.
- A dynamic loop (ϕ–*i*, blue line in Figure 3). This loop is directly measurable and comprises the static hysteresis loop plus the eddy losses.

**Figure 4:**
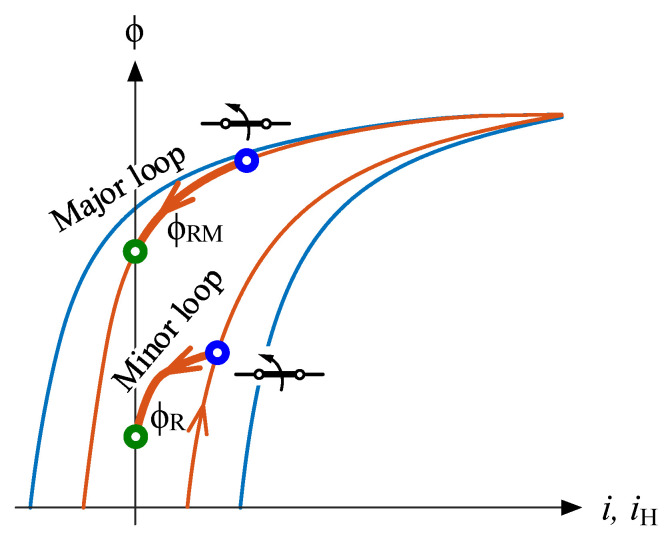
De-energization transient trajectories following a major and a minor loop and their respective residual fluxes.

The trajectories during the de-energization transient play a crucial role in minimizing the inrush current, given their dependence on residual flux. Figure 4 illustrates two representative trajectories of the de-energization transient.

When the switch aperture initiates between points 1 and 3 on Figure 3, for example, at the upper blue circle in Figure 4, the flux trajectory commences along the major loop. This trajectory continues until the residual flux consistently attains the maximum possible residual flux, denoted as ϕ_RM_. Conversely, if the aperture initiates at the lower blue circle, marked at a distinct de-energization point-on-wave, the flux follows an asymmetric minor loop trajectory. This trajectory persists until the residual flux reaches ϕ_R_, a value smaller than ϕ_RM_.

Determining the optimal moment to switch on the PV installation, a critical consideration for mitigating inrush current, is contingent upon the timing of the PV installation switch-off. This is because it influences the residual flux condition. Consequently, both switch-on and switch-off times are intended to be managed by the smart switch.

## 3. Optimizing Inrush Current Mitigation through Smart Switching

**Figure 5:**
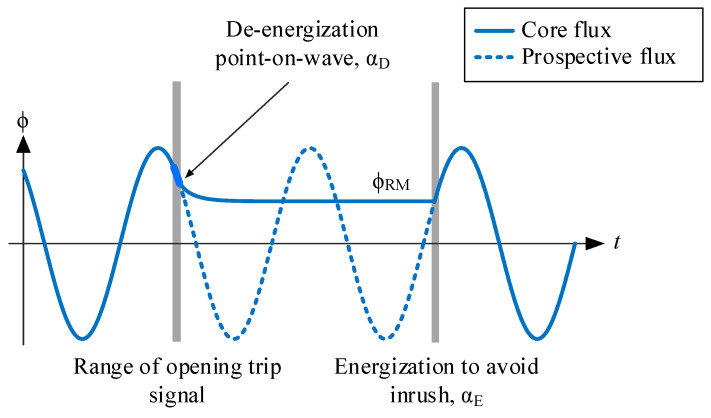
De-energization and energization strategy to avoid inrush current.

The fundamental approach to eliminating inrush currents is to ensure that the prospective flux at energization matches the residual flux. Thus, the ideal energization point-on-wave occurs when the prospective flux equals the residual flux, as illustrated in Figure 5. As it has been explained, the residual flux is solely determined by the de-energization trajectory, which, in turn, is influenced only by the de-energization point-on-wave. Therefore, the magnitude of the inrush current can be optimized by controlling both the de-energization and energization points-on-wave.

The proposed strategy is based on that previously presented in [50] and comprises two simple steps. Initially, it involves ensuring that the residual flux at de-energization attains its maximum value (ϕ_RM_). Subsequently, the second step entails energizing the transformer at the optimum point-on-wave for ϕ_RM_. The comprehensive strategy is summarized in Figure 5.

To enforce ϕ_RM_, the de-energization point-on-wave *α*_D_ must fall between 90° and α_RM_. The angle α_RM_ can be calculated using Equation (1), where ϕ_PEAK_ represents the maximum rated flux and *U*_1_ is the RMS primary transformer voltage.

(1) $$\alpha_{\mathrm{RM}}=180{}^{\circ}-\mathrm{asin}\left(\frac{\phi_{\mathrm{RM}}}{\phi_{\mathrm{PEAK}}}\right)\approx 180{}^{\circ}-\mathrm{asin}\left(\frac{\omega \phi_{\mathrm{RM}}}{\sqrt{2}U_{1}}\right)$$

The sudden interruption of current in a transformer with an IGBT breaker can induce significant overvoltages. To prevent this, de-energization is timed when the current approaches zero. This de-energization point-on-wave, denoted as *α*_0_, can be determined through Equation (2), where ϕ_0_ is the instantaneous flux when the current is null.

(2) $$\alpha_{0}=180{}^{\circ}-\mathrm{asin}\left(\frac{\phi_{0}}{\phi_{\mathrm{PEAK}}}\right)\approx 180{}^{\circ}-\mathrm{asin}\left(\frac{\omega \phi_{0}}{\sqrt{2}U_{1}}\right)$$

On the other hand, given the commonly low values of primary winding resistance and primary leakage inductance in PV installations, the expression for energization flux, detailed in Equation (3), is applicable.

(3) $$\phi =\phi_{R}+\frac{\sqrt{2}U_{1}}{\omega }\left[\sin\left(\omega t\right)-\sin\left(\alpha_{E}\right)\right]$$

This equation shows the influence of both the energization point-on-wave, denoted as α_E_, and the residual flux, denoted as ϕ_R_, on the flux during energization. To prevent subsequent inrush current, the offset in Equation (3) must be null. Consequently, if ϕ_R_ equals ϕ_RM_, the optimal α_E_ is determined by the following:

(4) $$\alpha_{E}=180{}^{\circ}-\mathrm{asin}\left(\frac{\phi_{\mathrm{RM}}}{\phi_{\mathrm{PEAK}}}\right)=\alpha_{\mathrm{RM}}$$

Therefore, the smart switch will perform the connection and disconnection of the photovoltaic installation at the time instances corresponding to the optimal points-on-wave (α_0_ and α_E_).

## 4. FPGA-Based Smart Switching Implementation

The smart switching strategy could be implemented solely by acquiring the supply voltage signal, but a significant drawback arises in the determination of α_0_ and α_E_ using Equations (2) and (4). For this, previous knowledge of offline data, such as ϕ_RM_, ϕ_0_, ϕ_PEAK_, and *U*_1_, is necessary. These parameters are different for each transformer, and some of them can be obtained through testing involving the static hysteresis loop and dynamic loop of the transformer, requiring a previous transformer testing stage and pre-programming the smart switch. However, implementing such a procedure is impractical for an online system, as presented in this paper. In addressing this challenge, the system incorporates a learning mechanism and acquires two new signals: the primary current and the transformer secondary voltage.

Firstly, the determination of the de-energization point-on-wave (α_0_) is facilitated by identifying the angle of the supply grid when the current crosses zero in the positive direction. To achieve this, the FPGA-based processor within the system is equipped with both a zero-crossing detector and a phase-locked loop (PLL).

**Figure 6:**
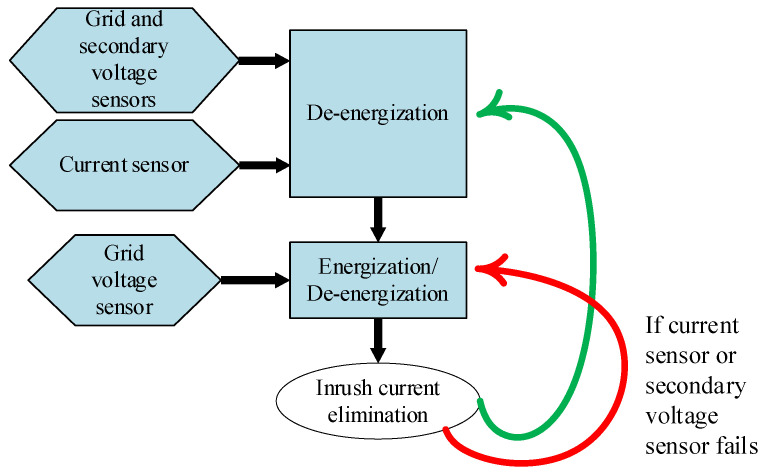
General scheme of the smart switching procedure, including possible sensor failures.

Precise determination of *α*_E_ requires knowledge of ϕ_RM_. To overcome this issue, the smart switching system acquires and integrates the secondary voltage before de-energization until several seconds after de-energization. This approach allows for the extraction of ϕ_RM_, facilitating the calculation of the corresponding energization point-on-wave *α*_E_. Subsequently, to ensure system resilience in the face of potential current and/or secondary voltage sensor failures, these calculated points-on-wave are stored in a read-only memory (ROM), which is valuable in case the primary current and secondary voltages are not available due to issues with the corresponding sensors. In this case, the system already has the necessary information (as long as the transformer or operating conditions do not change) to energize and de-energize the transformer during the appropriate moments by solely monitoring the grid voltage, as indicated in Figure 6.

In this way, the effectiveness of the proposed solution for inrush current elimination endures, even when confronted with current and voltage sensor issues. This resilience, which stems from the system’s capacity to learn and adapt based on information gleaned from the measurements, gives a high robustness to all the system.

**Figure 7:**
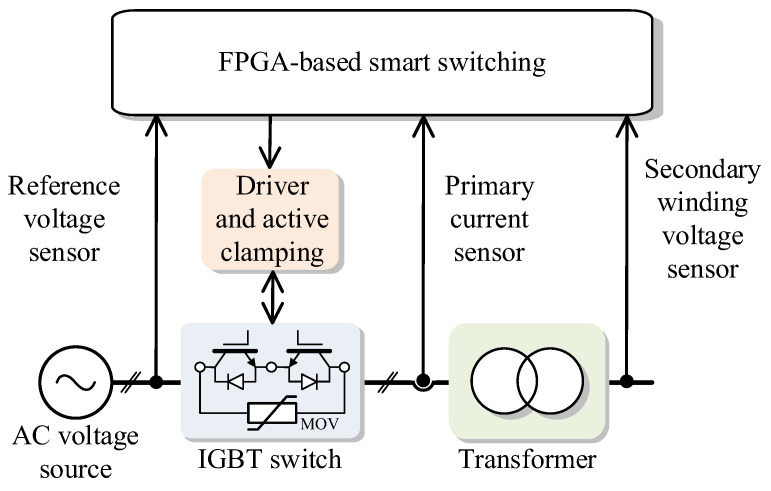
Schematic diagram of the proposed smart switch for inrush current optimization.

The circuit diagram of the proposed smart switch approach is presented in Figure 7. Both energization and de-energization are accomplished through the use of a semiconductor switch.

**Figure 8:**
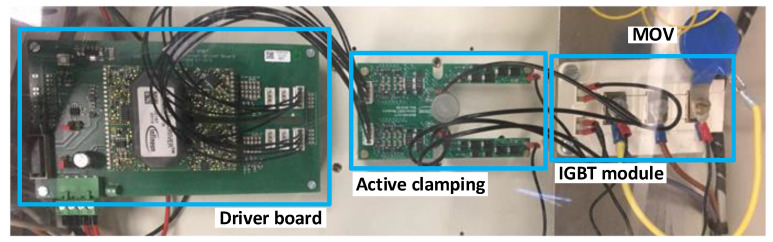
IGBT switch with driver and active clamping boards.

The semiconductor switch comprises two IGBTs, each equipped with an antiparallel diode, connected in series with a common emitter. It boasts a high chopping capability, ensuring a swift clearance time at any given instant, irrespective of the load nature, and notably, it operates without generating any electric arc. The IGBT used in this paper incorporates an active clamping feature to limit transient overvoltages when it turns off. A metal oxide varistor (MOV) is also connected in parallel with the breaker for the same purpose. The IGBT switch is shown in Figure 8.

**Figure 9:**
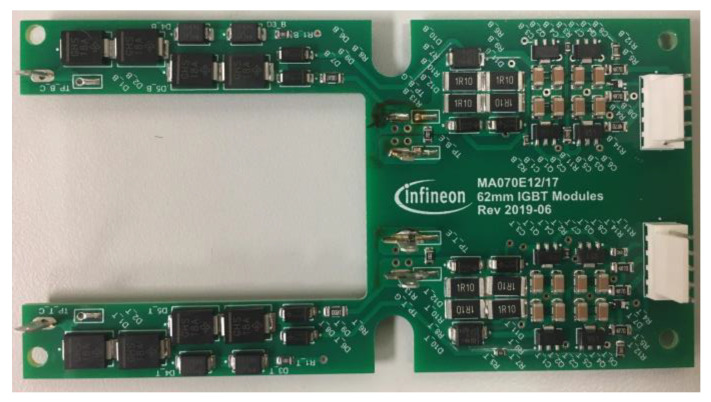
Detail of the active clamping board.

The conventional method for active clamping (Figure 9) is to use a chain of avalanche diodes connected between the collector and the gate of an IGBT. When the collector-emitter voltage exceeds the breakdown voltage of the diodes, the diode current sums up with the output current from the driver output. With the increased gate-emitter voltage, the transistor remains in active mode, causing the interruption of the turn-off process. This interruption slows down the speed, resulting in a limited overvoltage. Avalanche diodes conduct high peak currents while actively limiting overvoltage during this time period. The clamping diodes are directly connected to both the IGBT’s gate and the input of an amplifier on the same board. Consequently, the primary current source for recharging the gate is derived from the gate driver’s power supply rather than through the clamping diodes.

**Figure 10:**
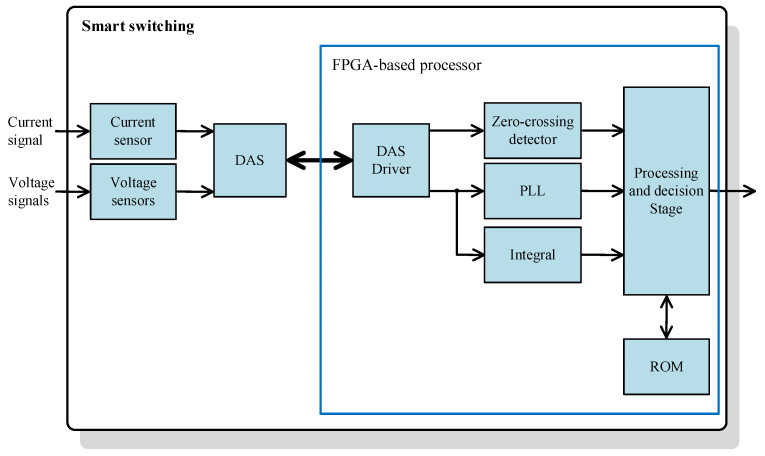
General structure of the proposed smart switching system.

Figure 10 illustrates the block diagram outlining the general architecture of the proposed smart switching system. The system delineates into three primary stages: primary sensors, a data acquisition system (DAS), and an FPGA-based processor.

The initial stage comprises primary sensors, featuring a current sensor (utilizing Hall Effect clamp meter technology) and two voltage sensors. The smart switch prototype, implemented as an FPGA-based processor, has been successfully developed on the dSPACE MicroLabBox platform, which integrates a Xilinx FPGA. Testing has been conducted using a 320 VA single-phase transformer.

The MicroLabBox integrates analog-to-digital converters boasting a 16-bit resolution, a sampling frequency of 1 million samples per second (sps), and an input range spanning from −10 V to +10 V. In this work, the measured signal has undergone internal resampling within the DSPACE system, resulting in a reduced sampling frequency of 8000 sps, deemed more suitable for digital systems. Prior to conversion, signal conditioning entails the use of a fully-differential isolation amplifier for electrical isolation and a low-pass anti-aliasing filter.

### FPGA-Based Processor

The processor has been fully implemented on an FPGA (Xilinx Kintex-7 XC7K325T, Xilinx, San Jose, CA, USA), and its development has been exclusively conducted using the Very High-Speed Integrated Circuit Hardware Description Language (VHDL) and IEEE standard libraries. It is noteworthy that no commercially available processing cores or libraries have been utilized in this development.

The FPGA-based processor serves as the ultimate stage within the system, responsible for issuing the trip signal to the IGBT switch. It is structured into two stages. The first stage encompasses three processing cores: a zero-crossing detector, an integrator core, and a PLL core. The second stage, receiving data from the preceding cores, determines the timing for dispatching both opening and closing trigger signals. Additionally, the FPGA-based processor incorporates a ROM and the essential drivers to ensure seamless communication with the DAS. It also integrates a corresponding finite state machine (FSM), imperative for handling the operation of all processing cores.

**Figure 11:**
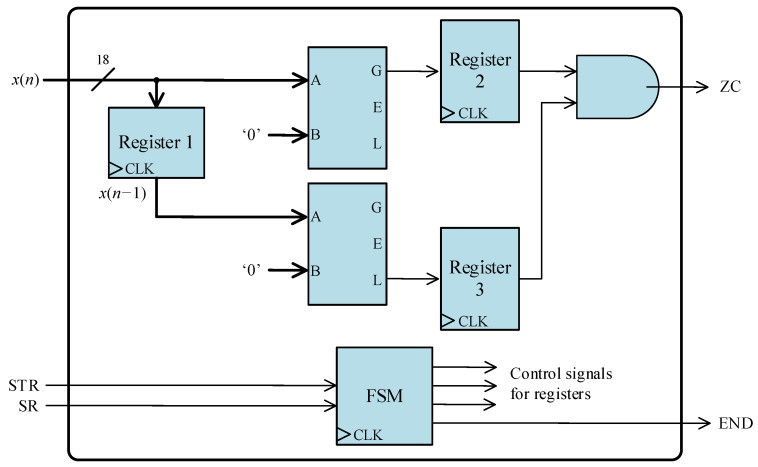
Architecture of the digital structure to achieve positive zero-crossing detection.

Figure 11 shows the fundamental architecture of the positive zero-crossing detector, employing two comparator blocks. The detection algorithm is straightforward: the two most recent input samples are compared to zero, and the positive zero-crossing is identified when the last sample is positive and the penultimate sample is negative.

The system encompasses three input signals: *x*(*n*), STR, and SR, along with two output signals, D2 and END. The transformer current, *x*(*n*), is an 18-bit signal presented in a 2.16 fixed-point format. STR serves as a 1-bit indicator signal, signaling the initiation of calculations, while SR, another 1-bit signal, informs the processing core of the availability of a new *x*(*n*) sample for reading. ZC is the output signal indicating the detection of a positive zero-crossing. Lastly, END is a 1-bit signal that indicates the completion of a calculation, indicating that a new result is ready for reading.

The processing core employs a parallel register (Register 1) to store the preceding input sample, *x*(*n* − 1). Upon the availability of a new sample at the input *x*(*n*), the register is enabled, facilitating the storage of the latest sample while discarding the penultimate one. Additionally, two registers are employed to regulate the output result flow. The core incorporates a finite state machine (FSM) to govern the activation of registers and, consequently, the data flow. This FSM also manages the indicator signals (STR, SR, and END).

The ZC signal is activated when both inputs of the AND gate are set to ‘1’, signifying the fulfillment of the specified conditions necessary for detecting a positive zero-crossing.

**Figure 12:**
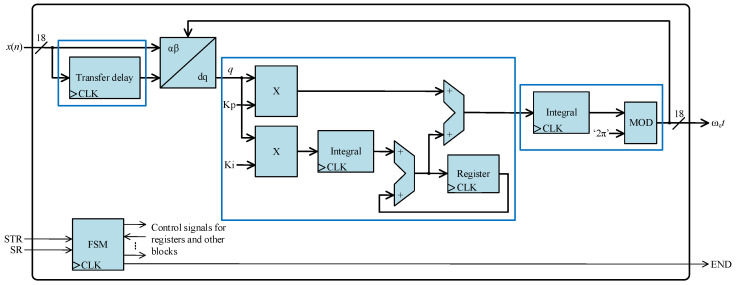
Architecture of the digital structure of the PLL core.

Figure 12 shows the overarching architecture of the PLL core. This core operates as a transfer delay PLL (TD-PLL). The TD-PLL method generates quadrature signals by introducing a delay to the original signal equivalent to a quarter of its frequency period (*T*/4).

The TD-PLL features a *T*/4 delay block for the generation of orthogonal signals (*x*_α_, *x*_β_). Through the application of the Park transformation, these variables undergo conversion to a rotating dq reference frame. The requisite phase for this transformation is derived from the PLL phase output (ω_e_*t*), ensuring that the *q* component is proportionally related to the phase error between the actual phase of the input signal and the estimated phase. This error is subsequently directed to a filter, a proportional-integrator (PI) controller, aiming to minimize it to zero and thereby achieving effective synchronization of the PLL phase output with the input phase.

Applying the Park transformation to *x*_α_ and *x*_β_, the *q* component is derived as follows:

(5) $$q=-x_{\alpha }\cdot \cos\left(\omega_{e}t\right)+x_{\beta }\cdot \sin\left(\omega_{e}t\right)$$

The sine and cosine functions used in the Park transformation are implemented in the FPGA through the utilization of lookup tables (LUTs).

**Figure 13:**
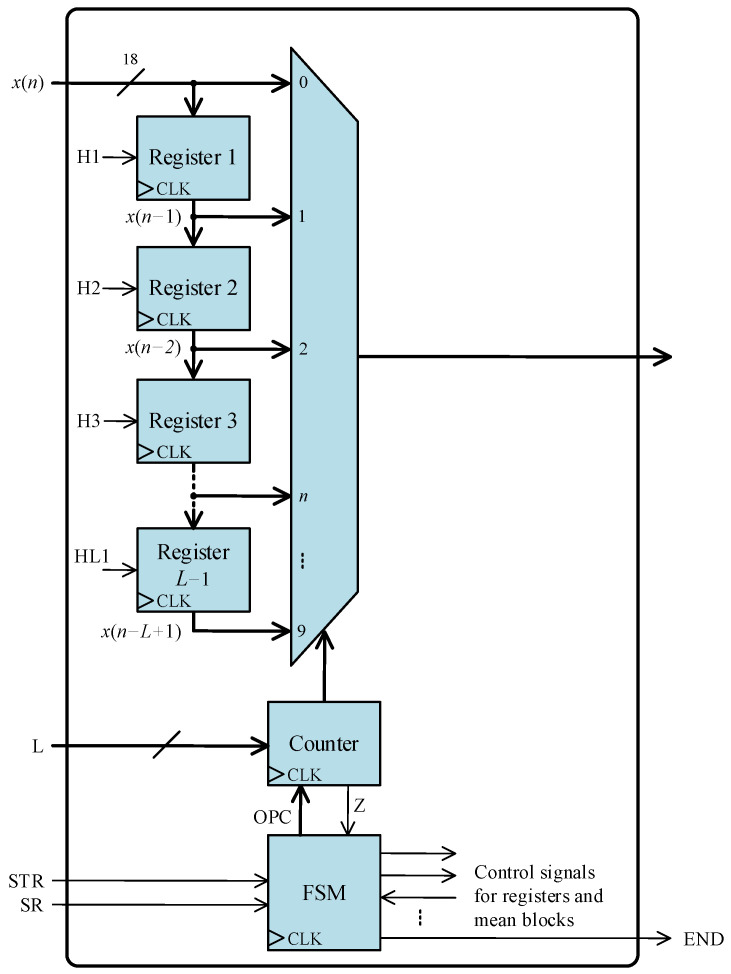
Architecture of the transfer delay block.

Figure 13 depicts the architecture of the transfer delay block. This block also has the STR and SR input signals and the END output signals, similar to the zero-crossing detection core. *L*−1 parallel registers are connected in cascades to store the *L*−1 most recent input samples, where *L* is equal to *T*/4. The initial outputs of all registers are equal to zero. The input *x*(*n*) and the registers’ outputs are connected through a multiplexor to the output. With the help of the multiplexor and a counter, the flow of present and past input samples can be controlled by the FSM.

It is noteworthy that the used FPGA operates at a base frequency of 100 MHz, significantly exceeding the sampling frequency.

**Figure 14:**
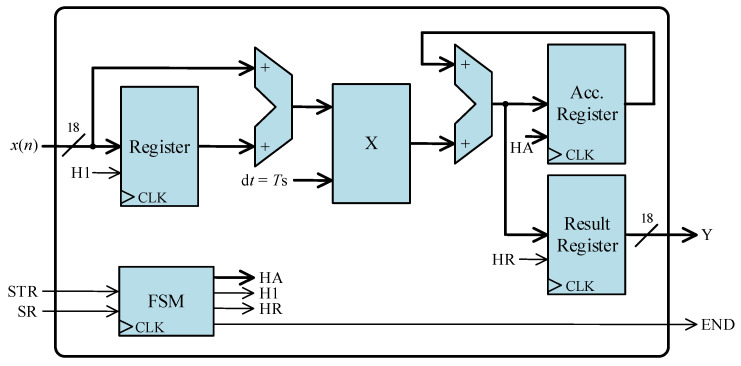
Architecture of the digital structure for computing the integral.

Figure 14 illustrates the architecture of the final processing core responsible for computing the integral (integral calculation being a component of the PLL) of the secondary voltage using the trapezoidal rule. Featuring a register at the input *x*(*n*), this processing core stores the previous sample, adds it to the current sample, and subsequently multiplies the result by the sampling period *T*_s_, corresponding to half of the sampling period, (*t*(*n*) − *t*(*n* − 1))/2. The cumulative integral at any given time is derived through successive summations facilitated by an accumulator.

## 5. Experimental Results

The efficacy of the smart switching system has been substantiated through validation with a 320 VA single-phase transformer characterized by a 120/72 V rating and short-circuit reactance values of 0.046 pu and 0.07 pu. In Figure 14, the associated waveforms depict the supply voltage, the transformer flux (derived through the integration of the secondary voltage), the transformer current, and the transformer secondary voltage.

**Figure 15:**
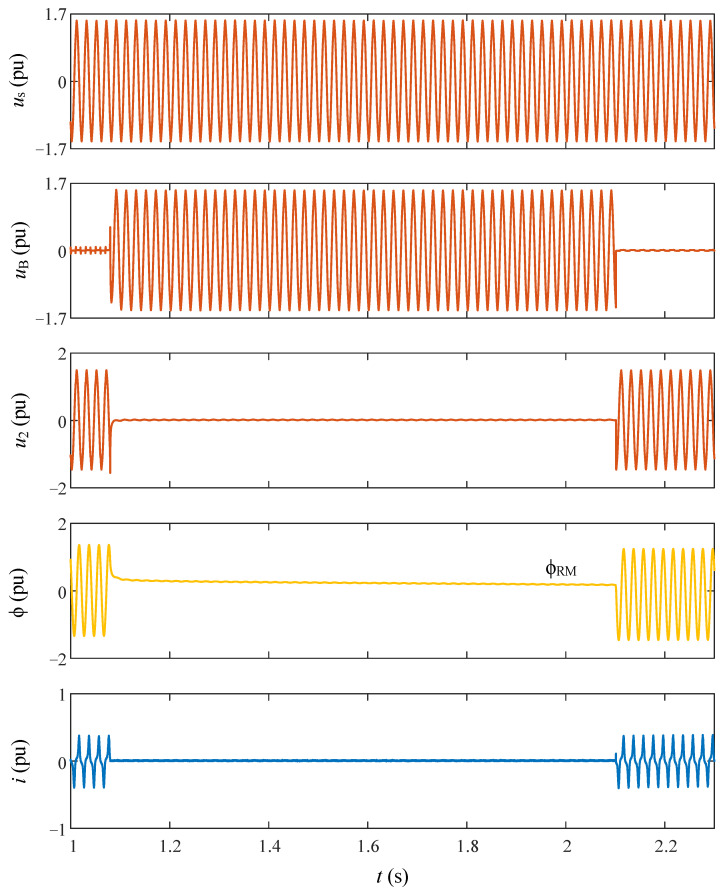
Experimental waveforms obtained during de-energization and re-energization of the transformer with smart switching.

**Figure 16:**
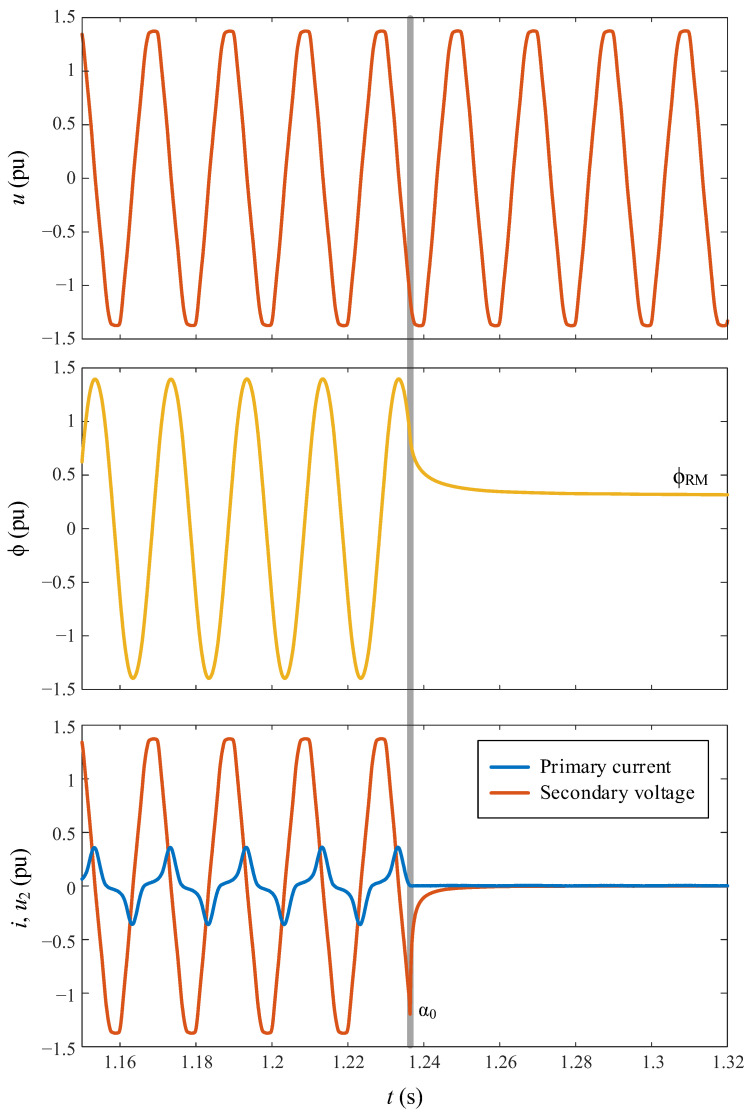
Experimental waveforms obtained during de-energization of the transformer.

The smart switching system requires only two sets of data: ϕ_RM_ and ϕ_0_, or their corresponding voltage points-on-wave α_RM_ and α_0_, coupled with an understanding of the utilized switching technology. As explained before, these data sets can be acquired through preliminary transformer no-load tests or by monitoring the primary current and secondary voltage of the transformer during the initial de-energization. All the signal waveforms acquired during the smart switching are depicted in Figure 15, while in Figure 16, they are depicted with more detail only during the de-energization.

As illustrated in Figure 16, the accurate de-energization point-on-wave can be determined by detecting the current positive zero-crossing, and ϕ_RM_ can be derived from the flux waveform after de-energization. Notably, in the event of a failure in either the current sensor or the sensor responsible for acquiring the secondary voltage, the proposed smart switching system can seamlessly persist in an operation without any compromise to its efficiency.

**Figure 17:**
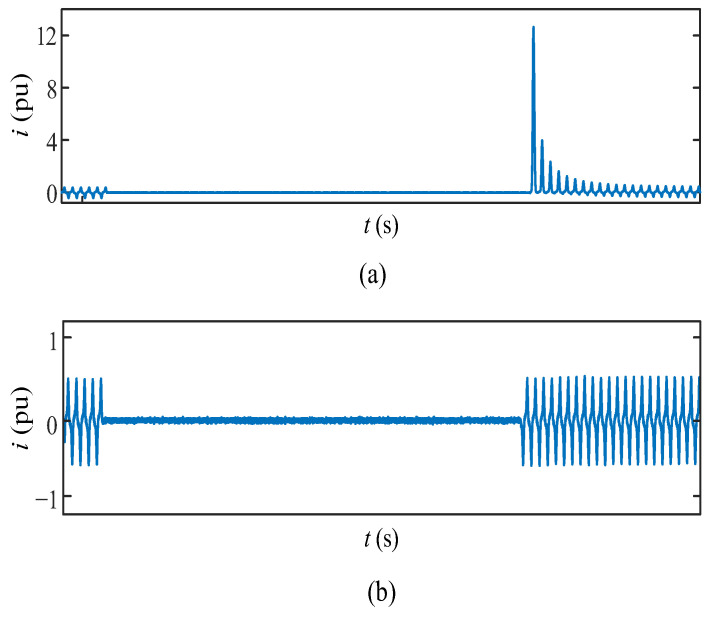
(**a**) Experimental inrush current resulting from random switching; (**b**) experimental current resulting with smart switching.

Figure 17 illustrates various levels of inrush currents. In Figure 17a, the worst-case scenario is depicted, showcasing a peak inrush current of approximately 12 pu. This extreme condition is realized with a de-energization point-on-wave (α_D_) at 90° and an energization point-on-wave (α_E_) at 270°. It is crucial to note that for larger transformers, the maximum residual flux values are higher (around 0.7 pu), potentially leading to more severe inrush currents with this conventional approach. In contrast, Figure 17b displays the resulting currents with the proposed smart switching system, demonstrating the absence of any overcurrent issues.

In future research, the proposed smart switching approach can be seamlessly extended to cater to three-phase PV systems, requiring a thorough analysis of the necessary modifications.

## 6. Conclusions

Uncontrolled inrush currents bring forth considerable risks, including heightened stress on equipment, potential transformer damage, and disruptions to the electrical grid. In the context of grid-connected PV systems, the significance of this issue cannot be overstated. A seamless and controlled energization process is indispensable for ensuring optimal performance, reducing equipment wear, and upholding the overall reliability of the power distribution network.

This paper presents the implementation of a novel FPGA-based smart switch designed to address the specific challenge of inrush currents in grid-connected PV systems. Taking advantage of the versatility and cost-effective reconfigurability of FPGAs, this proposed intelligent control system proves to be a suitable solution. It deftly navigates the complexities of residual flow estimation and provides robust functionality even in the presence of challenges with current and voltage sensors. Since the strategy only needs two pieces of information that never change, the system can allow for current sensor or secondary voltage failures and operate by simply monitoring the grid voltage. The system’s adaptive learning and storage capabilities contribute significantly to its overall resilience and effectiveness.

## Data Availability

Data are contained within the article.
